# Crystal in Iran: methamphetamine or heroin kerack

**DOI:** 10.1186/2008-2231-21-22

**Published:** 2013-03-15

**Authors:** Zahra Alam Mehrjerdi

**Affiliations:** 1 Iranian National Center for Addiction Studies (INCAS), Tehran University of Medical Sciences, Tehran, Iran

## Abstract

In recent years, methamphetamine use has dramatically increased in Iran while there is a crucial misunderstanding about the colloquial words related to methamphetamine among health providers, policy makers, clinicians, scholars and people in the community. The word Crystal refers to methamphetamine in some parts of Iran while in some other parts of the country, Crystal refers to a high purity street-level heroin which is called Kerack and its abuse is epidemic. Methamphetamine and heroin Kerack are different drugs in Iran. Methamphetamine is a stimulant drug while heroin Kerack is an opioid. Health providers especially clinicians and emergency medicine specialists should consider colloquial words that Iranian drug users apply. Special training courses should be designed and implemented for clinicians in Iran to inform them about methamphetamine and its frequently used colloquial words in the community. This issue has important clinical and health implications.

## To the Editor

Using opiates has a long history in Iran [1]. Opium and its pharmacological and psychotropic effects were known for several thousand years among Iranians [2] especially for managing general medical conditions such as pain, colic problems, headaches, and implementing anesthesia [3] but methamphetamine (MA) is a new psychostimulant drug and its abuse has recently surged in popularity [4,5] especially among young individuals in Iran [6]. Methamphetamine is available in different forms such as pure crystalline hydrochloride salt [7]. The main routes of methamphetamine administration include smoking, sniffing, injection and ingestion [7].

In Iran, smoking is the most common route of methamphetamine administration. Different medical treatments such as Bupropion, Modafinil, Dextroamphetamine, and Naltrexone have been examined for methamphetamine use treatment [8] but there is no pharmacological therapy with established efficacy for the treatment of this addictive disorder, nor is there any medication approved by the regulatory authorities for such treatment [9]. Cognitive**-**behavioral therapies including the Matrix Model are the main methods for treating methamphetamine use [10]. Recently, the transition from the traditional patterns of using opium and opium residues to methamphetamine smoking and/or injection has changed in to a new health concern in Iran [11].

A recent survey on 843 Iranian bodybuilders showed that 13.3% reported a history of amphetamine use [12]. There is evidence that methamphetamine use has negatively influenced opioid**-**dependent patients in methadone maintenance therapy. A recent study at the methadone maintenance treatment clinic of Baharan psychiatry hospital in Zahedan, Iran showed that methamphetamine use increased among patients from 6% in 2009 to approximately 20% in 2011 [13]. Methamphetamine use could also contribute to spreading viral infections such as HIV and HCV among injecting drug users in Iran [14]. The prevalence of methamphetamine use in Iran has dramatically resulted in emerging methamphetamine**-**associated psychosis and intoxication [15,16]. There is evidence that methamphetamine use has also imposed a crucial medical burden on the health sector of Iran [17]. As a highly addictive psychostimulant drug, the current increasing prevalence of methamphetamine use in Iran is in part due to its somewhat easy and cost**-**efficient syntheses in clandestine laboratories with inexpensive ingredients [11].

Methamphetamine is colloquially called Shisheh, Shabu, Dar va Panjereh, Gach, Lachaki, Ice, and Crystal in Iran. Shisheh is used as the most frequently used colloquial name for methamphetamine among drug users in many parts of Iran but using the name Crystal has widely implicated a crucial misunderstanding between the word methamphetamine and a common street**-**level heroin (e.g. Iranian Kerack, not to be mistaken with Crack Cocaine) in clinical and therapeutic settings, among health providers, policy makers, scholars and in the community. In Iran, drugs which have been named by drug users have sometimes similar western names, but they have different chemical compositions [18,19]. In some parts of Iran such as Khorasan province, Crystal widely refers to methamphetamine which is an addictive psychostimulant drug and its acute use increases alertness, attention, energy and decreases anxiety [20,21].

Acute methamphetamine use results in a number of other effects on the sympathetic nervous system, including hyper**-**tension, hyperthermia, increased breathing rate, constriction of blood vessels, tachycardia, and dialed pupils [22] but chronic methamphetamine use and abuse result in severe psychosis [17]. A recent study on fifty samples of methamphetamine obtained from antinarcotics police seized in Iran showed that the amounts of methamphetamine hydrochloride content in the samples were 33%-95% [23]. In other parts of Iran, the word Crystal widely refers to Iranian Kerack which its chemical composition varies not only with methamphetamine content, but also in the adulterants added to cut heroin. Iranian Kerack contains diacetylmorphine, acetylcodeine, 6**-**monoacetylmorphine, caffeine, papaverine, noscapine, dextromethorphan, morphine, codeine, phenobarbital and diazepam [24].

It should be noted that using Crack Cocaine is not prevalent in Iran and the word Kerack only refers to Iranian heroin Kerack not Crack Cocaine. The current misunderstanding is not limited to the community but it is also common among clinicians, emergency medicine specialists and scientific papers that have been recently published on the emergence of methamphetamine use problem by Iranian scholars. This issue has not received scientific attention yet. Methamphetamine use treatment is a current medical priority in Iran, and therefore, exploring precise names for methamphetamine is partly essential in the procedure of diagnosis, designing and implementing appropriate treatment and prevention programs for this group of illicit drug users. For clinicians, health providers, clinical toxicologists and researchers who work with methamphetamine users, awareness of the exact meanings of the present methamphetamine**-**related names in the community is essential. This issue is also important in critical situations including emergency department settings of hospitals where an immediate differential diagnosis of a type of drug used is an urgent medical priority to manage a drug**-**related situation such as severe methamphetamine intoxication or psychosis. This issue could play an important role in providing an appropriate pharmacological intervention.

For example, a recent study in the emergency department setting of Loghman Hakim Hospital in Tehran showed that severe dyspnea, sinus tachycardia with tachypnea, and echocardiogram, severe systolic dysfunction and heart failure were prevalent among methamphetamine**-**using patients [25]. Because of crucial health implications, clinicians and health providers should take in to consideration the types of drugs abused according to the accurate individual meanings that each patient reports. Special training courses should be designed and implemented by health policy makers to inform clinicians and health professionals about common colloquial methamphetamine**-**related words among Iranian drug users. This strategy has been never implemented in Iran and needs special attention in designing and tailoring drug use treatment programs for this newly emerged group of drug users in the country.
